# The C-reactive protein-albumin-lymphocyte index: a novel biomarker for metabolic dysfunction-associated fatty liver disease across three ethnic cohorts

**DOI:** 10.3389/fpubh.2026.1737437

**Published:** 2026-04-10

**Authors:** Yaojian Shao, Yusheng Zhang, Lulu Yin, Jianhua Yin, Yifan Lin

**Affiliations:** 1 Department of Gastroenterology, Taizhou Central Hospital (Taizhou University Hospital), Taizhou, Zhejiang, China; 2 Department of Laboratory Medicine, Naval Medical Centre, Naval Medical University, Shanghai, China; 3 Department of Endoscopy Center, Taizhou Central Hospital (Taizhou University Hospital), Taizhou, Zhejiang, China; 4 Department of Epidemiology, Naval Medical University, Shanghai, China

**Keywords:** CALLY index, inflammation, metabolic dysfunction-associated fatty liver disease (MAFLD), NHANES, nutrition, UK Biobank

## Abstract

**Background:**

Metabolic dysfunction-associated fatty liver disease (MAFLD) has emerged as a widespread chronic hepatic disorder worldwide. Early identification of individuals at elevated likelihood of MAFLD is crucial for preventing disease progression. The C-reactive protein-albumin-lymphocyte (CALLY) index, a novel composite biomarker reflecting systemic inflammation, nutritional status, and immune competence, offers potential utility in risk stratification. This study analyzed the relationship between the CALLY index and MAFLD prevalence utilizing cross-sectional data from three ethnically diverse cohorts in the U.S., the U.K and China.

**Methods:**

This cross-sectional study employed data from the US NHANES (2017–2020), the UK Biobank, and a Chinese hospital cohort. MAFLD was diagnosed via ultrasound-based controlled attenuation parameter (CAP) or the fatty liver index (FLI). The CALLY index was calculated from serum albumin, lymphocyte count, and CRP, and was natural log-transformed (ln-CALLY). Its association with prevalent MAFLD was assessed using multivariable logistic regression to estimate odds ratios (ORs) and 95% confidence intervals, with restricted cubic splines (RCS) and subgroup analyses used to evaluate robustness and potential non-linearity.

**Results:**

In NHANES, a per standard deviation (SD) increment in ln-CALLY was inversely associated with the odds of having MAFLD, corresponding to 24% lower odds (OR = 0.76, 95% CI: 0.64–0.90, *p* = 0.009). Similarly, in the UK Biobank cohort, each SD increase in ln-CALLY corresponded to 33% lower odds of MAFLD (OR = 0.67, 95% CI: 0.67–0.68, *p* < 0.001). A more pronounced risk reduction was observed in the Chinese population, where each SD rise in ln-CALLY was linked to 40% lower odds of MAFLD (OR = 0.60, 95% CI: 0.51–0.71, *p* < 0.001). The RCS analysis confirmed a non-linear inverse correlation between ln-CALLY and the odds of MAFLD, validating the dose-response protection identified through logistic regression. The area under the curve (AUC) of the ROC curve was 0.650 (95% CI: 0.636–0.665), indicating modest discriminative ability for identifying prevalent MAFLD.

**Conclusion:**

The CALLY index demonstrates an inverse relationship with MAFLD prevalence. This biomarker may help identify individuals with a higher likelihood of MAFLD, which could inform screening and risk assessment in clinical and population settings.

## 1 Introduction

Metabolic dysfunction-associated fatty liver disease (MAFLD), previously known as non-alcoholic fatty liver disease (NAFLD), is a serious metabolic disorder. Its core pathology is excessive hepatic lipid accumulation. Diagnostic confirmation of MAFLD requires histological evidence of hepatic steatosis (≥5% hepatocyte fat accumulation) concurrent with at least one metabolic criterion: elevated body mass index (BMI) signifying overweight/obesity, established type 2 diabetes mellitus (T2DM), or laboratory/clinical indicators of metabolic dysregulation. Unlike NAFLD, this diagnostic criterion applies regardless of whether the patient has a history of alcohol consumption or is concurrently suffering from other liver diseases (1). MAFLD encompasses a spectrum of liver pathologies, including simple steatosis, non-alcoholic steatohepatitis (NASH), and fibrosis (2). While the initial phase of MAFLD is typically benign, undetected and unmanaged progression may culminate in profound hepatic injury with potential advancement to hepatocellular carcinoma (HCC) (3). Furthermore, recent studies indicate that MAFLD also increases the risk of cardiovascular diseases and colorectal cancer (4, 5). In recent years, over 30% of the global population has been affected by this disease, and this figure is expected to continue rising with the enhancement of living conditions (2). People have realized that the key to preventing its progression lies in the timely identification of individuals more likely to have MAFLD for early intervention and treatment.

Research has established that chronic inflammation, compromised nutritional homeostasis, and dysregulated immune surveillance are determinants that modulate MAFLD pathogenesis and disease trajectory (6). Previous studies found that CRP, produced under the regulation of the inflammatory cytokine IL-6, can upregulate nuclear factor κB (NF-κB) activity; activated NF-κB can then interfere with insulin signaling transduction, thereby promoting MAFLD progression (7, 8). Albumin, a liver-synthesized plasma protein, serves as an established nutritional biomarker essential for sustaining protein homeostasis and physiological adaptability. Decreased albumin levels reflect potential underlying liver injury. Lymphocytes play a vital role in hepatic immune tolerance. As lymphocytic infiltration is a well-documented histopathological feature in NAFLD patients, this phenomenon implicates dysregulated T-cell responses as a pathogenic contributor to chronic hepatic disease progression (9, 10).

The C-reactive protein-albumin-lymphocyte (CALLY) index is an integrative biomarker calculated from serum CRP, albumin, and lymphocyte measurements, providing a multidimensional assessment of an individual's inflammatory activity, nutritional reserves, and immune competence. Currently, this index has emerged as a predictor of disease onset and prognosis in various conditions, including chronic obstructive pulmonary disease (11), ST-segment elevation myocardial infarction (12), and acute ischemic stroke (13). As for liver-related diseases, multiple studies have validated its role in assessing post-resection long-term outcomes of hepatocellular carcinoma and intrahepatic cholangiocarcinoma (14, 15). Compared with a single indicator, the evaluation dimensions of the CALLY index are more comprehensive. Furthermore, its relevant indicators are readily obtainable, giving it significant potential for clinical disease screening. However, evidence regarding the association between the CALLY index and MAFLD prevalence, particularly across diverse ethnic populations, remains scarce. This multi-cohort study aimed to fill this gap by comprehensively evaluating this relationship in three independent, ethnically diverse populations. Based on this background, we conducted this study using three independent cohorts from different countries, aiming to explore the relationship of the CALLY index with MAFLD and assess its potential for predicting this condition.

## 2 Methods

### 2.1 Data sources and study population

**Figure 1:**
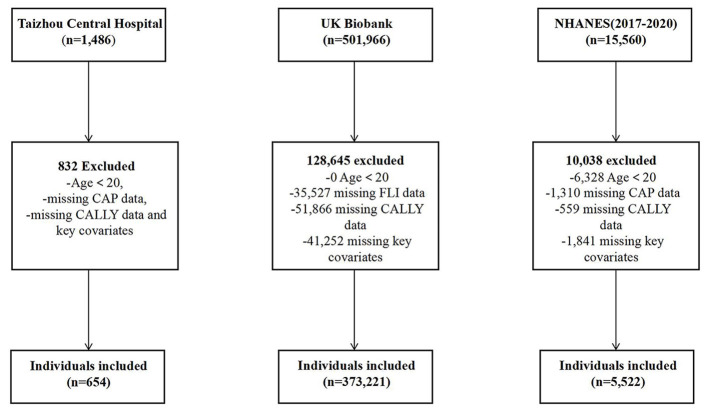
The flow diagram of the study participants. NHANES, national health and nutrition examination survey; CAP, controlled attenuation parameter; CALLY, C-reactive protein-albumin-lymphocyte; FLI, fatty liver index.

This study utilized data from three sources: the 2017–2020 cycles of the NHANES, the UK Biobank, and Taizhou Central Hospital, a tertiary hospital in Zhejiang, China. The NHANES is administered by the National Center for Health Statistics (NCHS) (16), constitutes a nationally representative cross-sectional surveillance system that collates multidimensional health metrics—including disease prevalence, nutritional biomarkers, dietary patterns, laboratory parameters, and clinical indicators—to evaluate population-level health and nutritional profiles in the U.S. The study was approved by the Ethics Review Committee of the National Center for Health Statistics (NCHS) and conducted in accordance with the Declaration of Helsinki. All participants have signed written informed consent forms. After screening, the final analytical sample comprised 5,522 participants. The UK Biobank constitutes a major biomedical database, which provides extensive genotypic and laboratory data from a comprehensive cohort of more than 500,000 individuals, facilitating a wide range of epidemiological investigations (17). The study was approved by the National Health Service (NHS) North West Center for Research Ethics Committee (11/NW/0382). All participants have signed written informed consent forms (project ID: 101971). After screening based on the inclusion and exclusion criteria, a total of 373,321 participants were enrolled in this study. We also reviewed the medical records of hospitalized patients at Taizhou Central Hospital (Taizhou, Zhejiang, China) between January 2023 and December 2024, and included 653 eligible patients who met the inclusion criteria in this study as external validation. The study protocol was approved by Taizhou Central hospital's Ethics Committee (Ethical approval number: 2026L-03-82). The complete participant selection flowchart for the above three cohorts is presented in Figure 1.

### 2.2 Definitions of the MAFLD and the CALLY index

The CALLY index was derived by the research team based on laboratory data obtained from NHANES. This composite biomarker was computed using the following equation: CALLY = [albumin concentration (g/L) × lymphocyte count (103 cells/μL)]/[C-reactive protein level (mg/L)/10] (18).

In NHANES (2017–March 2020), serum albumin was measured using a bromocresol purple (BCP) dye-binding colorimetric method on a Roche/Hitachi Cobas 6000 analyzer, and high-sensitivity C-reactive protein (hs-CRP) was quantified using a high-sensitivity immunoturbidimetric assay on the Roche Cobas 6000 (c501 module) according to NHANES laboratory protocols. In the UK Biobank, baseline serum albumin was measured by a colorimetric assay and high-sensitivity CRP by an immunoturbidimetric assay on Beckman Coulter AU5800 clinical chemistry analyzers, as described in the UK Biobank biomarker companion documentation. In the Taizhou Central Hospital cohort, serum albumin and hs-CRP were measured in the hospitals' central laboratory using routine automated chemistry/immunoassay platforms following internal and external quality-control procedures.

The diagnosis of MAFLD comprises two components. Firstly, one of the following three metabolic criteria must be met: (1) BMI ≥ 25 kg/m^2^, (2) Type 2 diabetes, or (3) at least two metabolic risk abnormalities. Metabolic risk abnormalities included: (i) Waist circumference (WC) (Men: ≥ 102 cm, Women: ≥ 88 cm), (ii) Hypertension (≥ 130/85 mmHg) or antihypertensive medication use, (iii) Triglycerides (TG) ≥ 150 mg/dl, (iv) HDL cholesterol (Men: < 40 mg/dl, Women: < 50 mg/dl), (v) Pre-diabetes (Fasting plasma glucose 100–125 mg/dl or Hemoglobin A1c 5.7%-6.4%), (vi) Homeostasis Model Assessment of Insulin Resistance Score (HOMA-IR) ≥ 2.5, (vii) High-sensitivity CRP (hs-CRP) plasma level > 2 mg/L. Secondly, the existence of hepatic steatosis must be unequivocally established. Ultrasound-based controlled attenuation parameter (CAP) measurements is a non-invasive technique that quantifies hepatic steatosis on a scale ranging from 100 to 400 dB/m, where elevated values correlate with increased liver fat accumulation (19). In line with established diagnostic criteria, a CAP cutoff value of 285 dB/m was employed as the threshold for identifying hepatic steatosis (20). Therefore, in the two cohorts of NHANES and Taizhou Central Hospital, fatty liver disease (FLD) was defined as the CAP measurement value that reached or exceeded this baseline value. However, there is a lack of liver imaging and histological data in the UK Biobank. Therefore, we adopted another reliable method, calculating the Fatty Liver Index (FLI) based on waist circumference, gamma glutamyl transferase, triglycerides, and body mass index (BMI), set FLI value higher than 60 as the diagnostic criterion for hepatic steatosis in the UK Biobank cohort to determine whether the subjects have liver steatosis (21, 22).

### 2.3 Covariates

To account for potential confounders in the CALLY-MAFLD relationship, we adjusted for multiple variables identified through previous studies. The analysis incorporated demographic factors (age, sex, and race), socioeconomic indicators (education level, family income-to-poverty ratio (PIR)/ Townsend deprivation index (TDI)), anthropometric measures (BMI), lifestyle factors (smoking history and alcohol use status), metabolic conditions (hypertension and diabetes presence), and biochemical markers (ALT, AST, and serum creatinine levels). Hypertension was defined by either self-reported diagnosis, a measured BP ≥140/90 mmHg, or current antihypertensive medication use. Diabetes mellitus was defined as a self-reported diagnosis, fasting glucose ≥126 mg/dl (7.0 mmol/L), HbA1c ≥6.5%, current glucose-lowering medication/insulin use or had International Classification Disease, version 10 (ICD-10) of E11 before the baseline assessment (23, 24). Due to different data sources, there may be slight differences in covariates among different cohorts. Race, serum creatinine levels and PIR/TDI was not included in the cohort of Taizhou Central Hospital.

### 2.4 Statistical analysis

Participant baseline characteristics were categorized based on MAFLD status (present vs. absent). Continuous data are reported as the means ± standard deviations, whereas categorical data are presented as proportions (%). Statistical analyses were performed to evaluate intergroup differences at baseline. In NHANES cohorts, sampling weights from the Mobile Examination Center (MEC) were incorporated to enhance the representativeness and robustness of the analytical results. Continuous variables with non-normal distributions, including the CALLY index, underwent logarithmic transformation (ln-CALLY) prior to analysis and were additionally stratified into quartiles (Q1–Q4) for categorical assessment. We evaluated ln-CALLY-MAFLD associations using adjusted logistic regression models, with Pearson's χ^2^test and Kruskal-Wallis test employed for initial group comparisons. The analysis employed three progressively adjusted models: Model 1, Model 2, which incorporated incorporating demographic and lifestyle factors (age, race/ethnicity, marital status, education, PIR/TDI, BMI category, smoking, and alcohol use), and Model 3, which additionally adjusted for clinical biomarkers (hypertension, diabetes, ALT, AST, and serum creatinine). Due to the cross-sectional design and the relatively high prevalence of MAFLD in our study populations, we acknowledge that odds ratios (ORs) from logistic regression may overestimate the strength of association compared to prevalence ratios. Nevertheless, we present ORs as the primary measure because they are the natural output of logistic regression—a robust and widely used method for binary outcomes—and they facilitate comparisons with existing literature. To ensure correct interpretation, we describe ORs as associations with the odds or prevalence of MAFLD, rather than as risk estimates. Potential non-linear associations between the ln-CALLY index and the odds of MAFLD were examined using restricted cubic spline regression with adjustment matching Model3′s covariates. Subgroup analyses stratified by age, sex, BMI category, smoking status, alcohol use, hypertension status, and diabetes status were conducted to examine potential variations in the CALLY-MAFLD association across different populations. Predictive accuracy of this index for identifying prevalent MAFLD was quantified by analyzing the receiver operating characteristic (ROC) curve of NHANES cohort and expressed as the area under the curve (AUC). All statistical computations were performed using R software (v.4.3.3). A two-sided *p* < 0.05 was considered statistically significant.

## 3 Results

### 3.1 Baseline characteristics of participants

As shown in Table 1, significant differences were observed between the MAFLD and non-MAFLD groups regarding age, sex, race/ethnicity, smoking history in all three cohorts. Regarding metabolic characteristics, the prevalence of obesity, diabetes and hypertension in MAFLD group increased significantly, accompanied by a significant increase in liver enzyme levels (*P* < 0.05). These results suggest that age, men, obesity, and metabolic syndrome were associated with prevalent MAFLD, while differences in race/ethnicity, smoking history may reflect genetic or environmental influences.

**Table 1:** Baseline characteristics of participants grouped by MAFLD status.

Characteristic	Overall	Non-MAFLD group	MAFLD group	*P*-value^b^
Characteristics of adults in NHANES				
Participants (%)	5,522 (100%)^a^	3,409 (61.73%)^a^	2,113 (38.26%)^a^	
Age (years)				< 0.001
< 40	1,701 (35.95%)	1,234 (40.91%)	467 (27.37%)	
40–60	2,041 (38.05%)	1,150 (35.34%)	891 (42.75%)	
>60	1,780 (25.99%)	1,025 (23.75%)	755 (29.88%)	
Sex (%)				< 0.001
Men	2,806 (49.81%)	1,599 (45.50%)	1,207 (57.29%)	
Women	2,716 (50.19%)	1,810 (54.50%)	906 (42.71%)	
Race (%)				< 0.001
Mexican American	662 (8.05%)	306 (5.89%)	356 (11.81%)	
Other Hispanic	543 (6.77%)	340 (7.02%)	203 (6.33%)	
Non-Hispanic White	2,128 (66.71%)	1,290 (67.44%)	838 (65.44%)	
Non-Hispanic Black	1,386 (10.05%)	947 (11.07%)	439 (8.27%)	
Other race	803 (8.42%)	526 (8.57%)	277 (8.17%)	
Education level (%)				< 0.001
Below high school	857 (9.07%)	498 (8.57%)	359 (9.92%)	
High school	1,330 (26.57%)	792 (24.44%)	538 (30.26%)	
Above high school	3,335 (64.37%)	2,119 (66.99%)	1,216 (59.81%)	
PIR	3.21 (1.62)	3.24 (1.64)	3.16 (1.59)	0.3
BMI (%)				< 0.001
< 30	3,092 (57.16%)	2,444 (73.94%)	648 (28.05%)	
≥30	2,430 (42.84%)	965 (26.06%)	1,465 (71.95%)	
Smoking status (%)				0.001
Current smoker	1,057 (17.44%)	689 (18.40%)	368 (15.78%)	
Former smoker	1,446 (27.76%)	781 (24.74%)	665 (33.00%)	
Never smoker	3,019 (54.80%)	1,939 (56.86%)	1,080 (51.22%)	
Drinking status (%)				0.086
No	1,172 (16.76%)	676 (15.83%)	496 (18.37%)	
Yes	4,350 (83.24%)	2,733 (84.17%)	1,617 (81.63%)	
Hypertension (%)				< 0.001
No	3,036 (61.56%)	2,139 (70.60%)	897 (45.89%)	
Yes	2,486 (38.44%)	1,270 (29.40%)	1,216 (54.11%)	
Diabetes (%)				< 0.001
No	4,415 (85.24%)	3,006 (92.68%)	1,409 (72.32%)	
Yes	1,107 (14.76%)	403 (7.32%)	704 (27.68%)	
ALT (U/L)	22.92 (17.39)	19.76 (15.16)	28.40 (19.52)	< 0.001
AST (U/L)	21.90 (12.64)	20.95 (12.28)	23.53 (13.09)	< 0.001
Serum creatinine (mg/dl)	0.88 (0.36)	0.87 (0.31)	0.89 (0.44)	0.2
ln-CALLY	6.12 (1.17)	6.36 (1.16)	5.71 (1.06)	< 0.001
Characteristics of adults in UK biobank				
Participants (%)	373,321 (100%)	226,110 (60.56%)	147,211 (39.43%)	
Age (years)				< 0.001
< 40	6 (0.00%)	3 (0.00%)	3 (0.00%)	
40–60	148,587 (39.80%)	86,426 (38.22%)	62,161 (42.23%)	
>60	224,728 (60.20%)	139,681 (61.78%)	85,047 (57.77%)	
Sex (%)				< 0.001
Men	173,978 (46.60%)	79,959 (35.36%)	94,019 (63.87%)	
Women	222,900 (53.77%)	146,151 (64.64%)	53,192 (36.13%)	
Race (%)				< 0.001
Asian	7,733 (2.07%)	4,806 (2.13%)	2,927 (1.99%)	
Black	5,682 (1.52%)	3,303 (1.46%)	2,379 (1.62%)	
Mixed	2,131 (0.57%)	1,374 (0.61%)	757 (0.51%)	
Other races	3,172 (0.85%)	1,884 (0.83%)	1,288 (0.87%)	
White	354,603 (94.99%)	214,743 (94.97%)	139,860 (95.01%)	
Education level (%)				< 0.001
High school or below	254,416 (68.15%)	146,192 (64.66%)	108,224 (73.52%)	
Above high school	118,905 (31.85%)	79,918 (35.34%)	38,987 (26.48%)	
TDI	−1.35 (3.06)	−1.51 (2.97)	−1.09 (3.17)	0.3
BMI (%)				< 0.001
< 30	279,707 (74.92%)	216,702 (95.84%)	63,005 (42.80%)	
≥30	93,614 (25.08%)	9,408 (4.16%)	84,206 (57.20%)	
Smoking status (%)				< 0.001
Current smoker	39,102 (10.47%)	22,369 (9.89%)	16,733 (11.37%)	
Former smoker	131,870 (35.32%)	72,005 (31.85%)	59,865 (40.67%)	
Never smoker	202,349 (54.20%)	131,736 (58.26%)	70,613 (47.97%)	
Drinking status (%)				0.095
No	15,909 (4.26%)	9,535 (4.22%)	6,374 (4.33%)	
Yes	357,412 (95.74%)	216,575 (95.78%)	140,837 (95.67%)	
Hypertension (%)				< 0.001
No	97,082 (26.00%)	77,506 (34.28%)	19,576 (13.30%)	
Yes	276,239 (74.00%)	148,604 (65.72%)	127,635 (86.70%)	
Diabetes (%)				< 0.001
No	350,701 (93.94%)	220,349 (97.45%)	130,352 (88.55%)	
Yes	22,620 (6.06%)	5,761 (2.55%)	16,859 (11.45%)	
ALT (U/L)	23.66 (14.15)	19.51 (9.70)	30.04 (17.21)	< 0.001
AST (U/L)	26.28 (10.11)	24.66 (8.16)	28.76 (12.12)	< 0.001
Serum creatinine (μmol/L)	72.56 (18.66)	70.00 (16.74)	76.50 (20.67)	< 0.001
ln-CALLY	8.68 (1.09)	8.94 (1.06)	8.28 (1.01)	< 0.001
Characteristics of adults in Taizhou Central Hospital				
Participants (%)	653 (100%)	455 (69.67%)	198 (30.32%)	
Age (years)				0.028
< 40	107 (16.39%)	64 (14.07%)	43 (21.72%)	
40–60	296 (45.33%)	206 (45.27%)	90 (45.45%)	
>60	250 (38.28%)	185 (40.66%)	65 (32.83%)	
Sex (%)				< 0.001
Men	318 (48.70%)	194 (42.64%)	124 (62.63%)	
Women	335 (51.30%)	261 (57.36%)	74 (37.37%)	
Education level (%)				0.127
Below high school	499 (76.42%)	357 (78.46%)	142 (71.72%)	
High school	86 (13.17%)	57 (12.53%)	29 (14.65%)	
Above high school	68 (10.41%)	41 (9.01%)	27 (13.64%)	
BMI (%)				< 0.001
< 28	584 (89.43%)	432 (94.95%)	152 (76.77%)	
≥28	69 (10.57%)	23 (5.05%)	46 (23.23%)	
Smoking status (%)				0.016
No	545 (83.59%)	390 (85.90%)	155 (78.28%)	
Yes	107 (16.41%)	64 (14.10%)	43 (21.72%)	
Drinking status (%)				0.055
No	595 (91.12%)	421 (92.53%)	174 (87.88%)	
Yes	58 (8.88%)	34 (7.47%)	24 (12.12%)	
Hypertension (%)				0.010
No	455 (69.68%)	331 (72.75%)	124 (62.63%)	
Yes	198 (30.32%)	124 (27.25%)	74 (37.37%)	
Diabetes (%)				< 0.001
No	466 (71.36%)	368 (80.88%)	98 (49.49%)	
Yes	187 (28.64%)	87 (19.12%)	100 (50.51%)	
ALT (U/L)	22.58 (18.27)	30.25 (24.94)	19.25 (13.15)	< 0.001
AST (U/L)	21.29 (10.65)	23.61 (13.59)	20.28 (8.90)	0.005
ln-CALLY	6.34 (1.33)	6.61 (1.26)	5.73 (1.27)	< 0.001

^a^*n* (unweighted) (%); Mean (SD).

^b^Pearson's χ^2^: Rao & Scott adjustment; Design-based Kruskal–Wallis test.

BMI, body mass index; PIR, family income-to-poverty ratio; ALT, alanine aminotransferase; AST, aspartate aminotransferase; CALLY, C-reactive protein-albumin-lymphocyte index; TDI, townsend deprivation index.

### 3.2 Relationship between the ln-**CALLY index and MAFLD**

**Figure 2:**
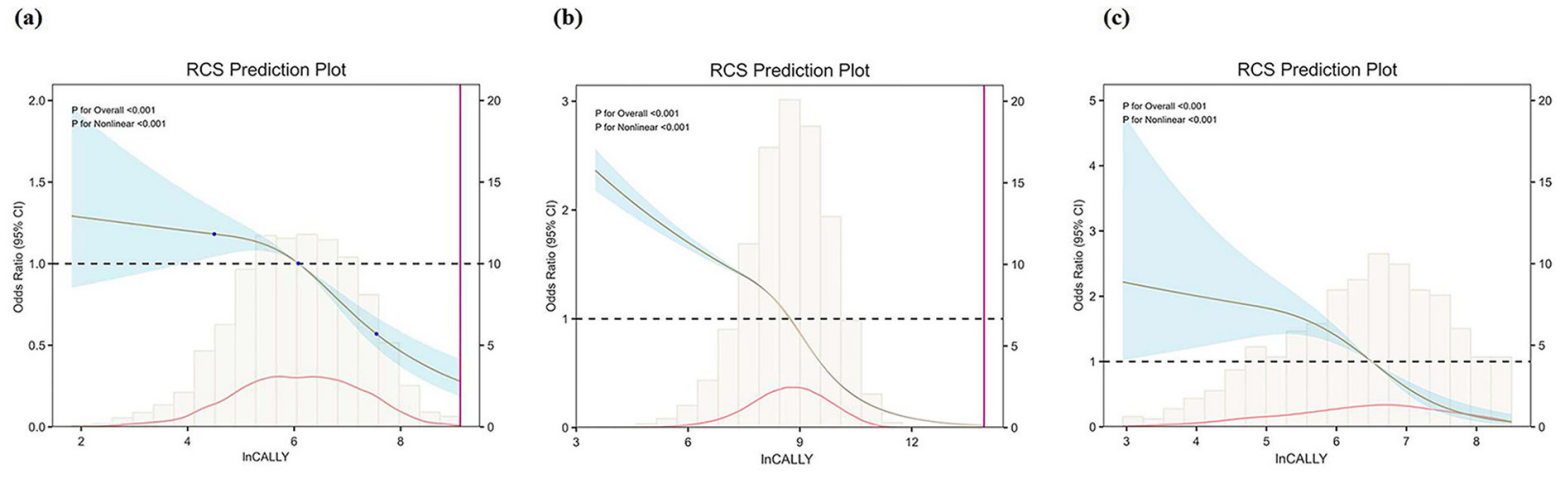
Restricted cubic spline plots illustrating a dose response between ln-CALLY index and the adjusted odds of MAFLD. The odds of MAFLD gradually decreases as ln-CALLY increases. **(a)** NHANES cohort; **(b)** UK Biobank cohort; **(c)** Taizhou Central Hospital cohort. MAFLD, metabolic dysfunction-associated fatty liver disease; CALLY, C-reactive protein-albumin-lymphocyte.

Table 2 presents logistic regression results demonstrating each 1-unit ln-CALLY increment was inversely associated with the odds of MAFLD (NHANES: OR = 0.60, 95% CI: 0.53–0.69; UK Biobank: OR = 0.55, 95% CI: 0.55–0.55, Taizhou Central Hospital: OR = 0.59, 95% CI: 0.52–0.68; all *P* < 0.001) in unadjusted analyses. To account for potential confounding factors, Models 2 and 3 were constructed. In Model 2 and Model 3, the strength of the association was attenuated but remained significant. The fully adjusted Model 3 confirmed that higher ln-CALLY was associated with lower odds of MAFLD (NHANES: OR = 0.76, 95% CI: 0.64–0.90, *P* = 0.009; UK Biobank: OR = 0.67, 95% CI: 0.67–0.68, *P* < 0.001; Taizhou Central Hospital: OR = 0.60, 95% CI: 0.51–0.71; *P* < 0.001). In Model 1, when analyzing ln-CALLY categorized into quartiles, using Q1 as the reference group, a decreasing trend in the odds of MAFLD was observed across Q2, Q3, and Q4, with the Q4 group (highest ln CALLY) showing particularly lower odds (NHANES: OR = 0.21, 95% CI: 0.14–0.31; UK Biobank: OR = 0.17, 95% CI: 0.16–0.17, Taizhou Central Hospital: OR = 0.12, 95% CI: 0.06–0.21; all *P* < 0.001). The decreasing trend in risk across quartiles persisted in Model 3, with significantly lower odds in Q3 (NHANES: OR = 0.56, 95% CI: 0.34–0.93, *P* = 0.035; UK Biobank: OR = 0.52, 95% CI: 0.50–0.53, *P* < 0.001; Taizhou Central Hospital: OR = 0.28, 95% CI: 0.16–0.49; *P* < 0.001) and Q4 (NHANES:OR = 0.43, 95% CI: 0.22–0.83, *P* = 0.027; UK Biobank: OR = 0.29, 95% CI: 0.28–0.30, *P* < 0.001; Taizhou Central Hospital: OR = 0.13, 95% CI: 0.06–0.26; *P* < 0.001) compared to Q1 (all *P* < 0.05). The analysis reveals a consistent inverse relationship between elevated ln-CALLY levels and MAFLD incidence, persisting across multiple covariate adjustments. This stability suggests ln-CALLY's potential as an independent biomarker inversely associated with hepatic steatosis. Furthermore, to examine a potential non-linear relationship, RCS curves were plotted to visualize the relationship between the ln-CALLY index and the odds of MAFLD. The results demonstrated a significant non-linear inverse relationship between ln-CALLY and the odds of MAFLD (all *P* for non-linearity < 0.001). As shown in Figure 2, the adjusted odds of MAFLD gradually decreases as ln-CALLY increases, which is consistent with the findings from the logistic regression analysis.

**Table 2:** Association between ln-CALLY and MAFLD.

Characteristic	Model 1			Model 2			Model 3		
	OR^a^	95% CI^a^	*p*-value	OR^a^	95% CI^a^	*p*-value	OR^a^	95% CI^a^	*p*-value
NHANES									
ln-CALLY	0.60	0.53, 0.69	**< 0.001**	0.74	0.64, 0.86	**< 0.001**	0.76	0.64, 0.90	**0.009**
ln-CALLY quartile									
Q1	—	—		—	—		—	—	
Q2	0.90	0.74, 1.10	0.277	0.94	0.73, 1.22	0.593	0.95	0.67, 1.36	0.699
Q3	0.41	0.29, 0.57	**< 0.001**	0.55	0.38, 0.79	**0.005**	0.56	0.34, 0.93	**0.035**
Q4	0.21	0.14, 0.31	**< 0.001**	0.40	0.25, 0.64	**0.002**	0.43	0.22, 0.83	**0.027**
UK biobank									
ln-CALLY	0.55	0.55, 0.55	**< 0.001**	0.66	0.66, 0.67	**< 0.001**	0.67	0.67, 0.68	**< 0.001**
ln-CALLY quartile									
Q1	—	—		—	—		—	—	
Q2	0.62	0.61, 0.63	**< 0.001**	0.76	0.74, 0.78	**< 0.001**	0.74	0.72, 0.76	**< 0.001**
Q3	0.37	0.36, 0.37	**< 0.001**	0.52	0.50, 0.53	**< 0.001**	0.52	0.50, 0.53	**< 0.001**
Q4	0.17	0.16, 0.17	**< 0.001**	0.27	0.27, 0.28	**< 0.001**	0.29	0.28, 0.30	**< 0.001**
Taizhou Central Hospital									
ln-CALLY	0.59	0.52, 0.68	**< 0.001**				0.60	0.51, 0.71	**< 0.001**
ln-CALLY quartile									
Q1	—	—		—	—		—	—	
Q2	0.78	0.50, 1.21	0.27	0.80	0.50, 1.29	0.36	0.80	0.49, 1.34	0.41
Q3	0.31	0.20, 0.50	**< 0.001**	0.31	0.19, 0.52	**< 0.001**	0.28	0.16, 0.49	**< 0.001**
Q4	0.12	0.06, 0.21	**< 0.001**	0.13	0.07, 0.24	**< 0.001**	0.13	0.06, 0.26	**< 0.001**

Model 1: no covariates were adjusted. Model 2: age, sex, race, education, PIR/TDI, BMI, smoking, and drinking were adjusted. Model 3: age, sex, race, education, PIR/TDI, BMI, smoking, drinking, hypertension, diabetes, ALT, AST and serum creatinine were adjusted. Race, serum creatinine levels and PIR/TDI was not included in the cohort of Taizhou Central Hospital.

OR^a^, odds ratio; CI^a^, confidence interval; BMI, body mass index; PIR family income-to-poverty ratio; ALT, alanine aminotransferase; AST, aspartate aminotransferase; CALLY, C-reactive protein-albumin-lymphocyte index; TDI, townsend deprivation index. Bold indicates statistical significance (*p* < 0.05).

### 3.3 Subgroup analysis

**Figure 3:**
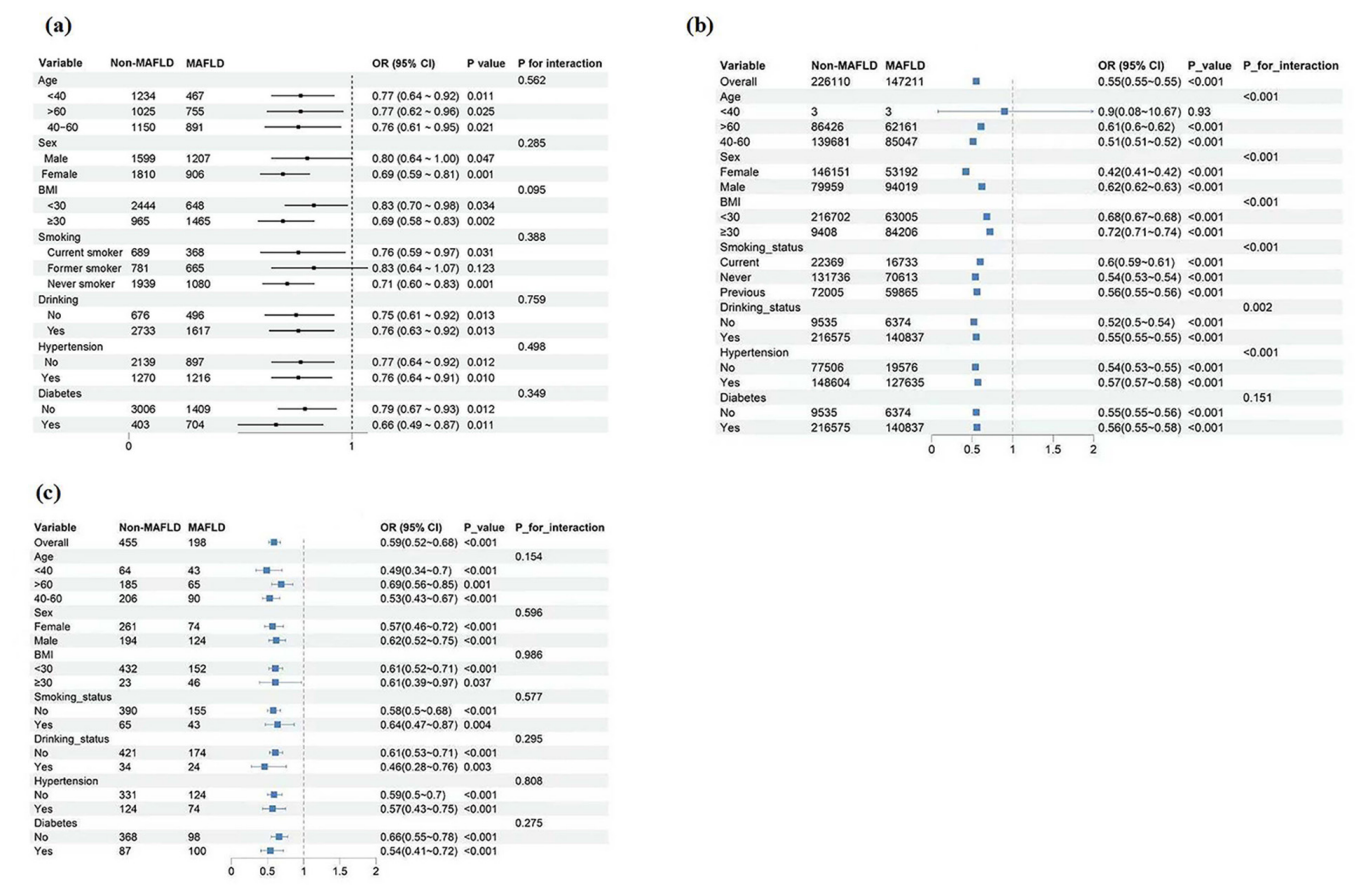
Subgroup analysis of the association between ln-CALLY and the odds of MAFLD. The robust inverse relationship remained stable across all examined subgroups, confirming the association's reliability. **(a)** NHANES cohort; **(b)** UK Biobank cohort; **(c)** Taizhou Central Hospital cohort. MAFLD, metabolic dysfunction-associated fatty liver disease; BMI, body mass index.

Subgroup analyses evaluated the consistency of the ln-CALLY-MAFLD association, revealing no significant effect modification by age, sex, BMI category, smoking status, alcohol use, hypertension, or diabetes status in NHANES and Taizhou Central Hospital cohorts (all interaction *P*-values >0.05). The robust inverse relationship remained stable across all examined subgroups, as illustrated in Figure 3, confirming the association's reliability regardless of population characteristics. In contrast, analyses in the UK Biobank cohort revealed significant effect modification by age, sex, BMI, hypertension, smoking, and drinking status (all interaction *P*-values < 0.05), indicating notable heterogeneity in the strength of the association across these subgroups. This heterogeneity could be attributable to the unique characteristics of the UK Biobank population, which is pre-dominantly composed of British individuals aged 40 above, a demographic that might exhibit distinct susceptibility patterns. Crucially, despite this heterogeneity in the magnitude of the effect, the inverse association (OR < 1) between ln-CALLY and MAFLD was consistently observed across all subgroups, indicating a stable direction of association toward lower odds of MAFLD.

### 3.4 ROC analysis

**Figure 4:**
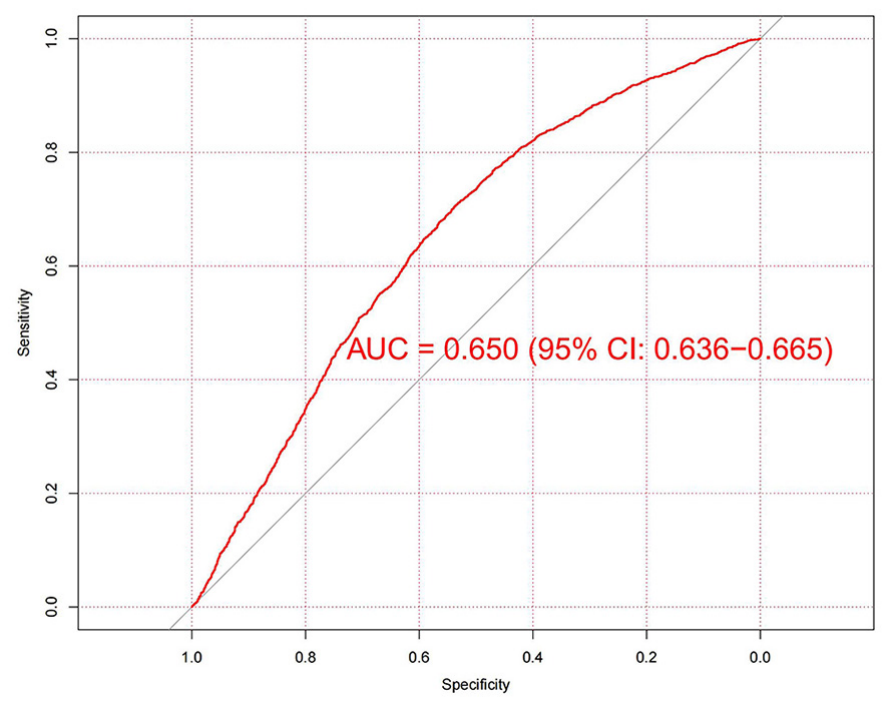
Receiver operating characteristic analysis for identifying MAFLD using the ln-CALLY index in the NHANES cohort. The derived area under the curve indicating some predictive value for MAFLD identification.

Figure 4 displays the receiver operating characteristic (ROC) curve analysis for identifying prevalent MAFLD utilizing the ln-CALLY index in NHANES cohort. The derived area under the curve (AUC) value is 0.650 (95% CI: 0.636–0.665), indicating some predictive value for MAFLD identification.

## 4 Discussion

With documented year-on-year increases in incidence, MAFLD has now become a serious metabolic disorder requiring systematic clinical engagement. Although its pathogenesis has not yet been fully elucidated, ectopic lipid deposition is currently considered a major trigger for hepatic steatosis, which evolves into sustained inflammation, culminating in parenchymal damage, NASH development, and progressive fibrogenesis (25). MAFLD progression is strongly influenced by three key factors: systemic inflammation, nutritional status, and immune regulation. Central to this process is hepatic inflammation, which drives disease advancement through cytokine-mediated disruption of insulin signaling pathways. The IKK/NF-κB pathway serves as a primary inflammatory mediator, with adipocyte-derived cytokines (TNF-α, IL-1β, and IL-6) activating both intracellular kinases and hepatic CRP production, creating a vicious cycle of inflammation and metabolic dysfunction. CRP, produced in response to upstream cytokine signals, primarily IL-6, can upregulate NF-κB activity; the activated NF-κB can then interfere with insulin signaling transduction, further contributing to the onset and progression of MAFLD (8, 26). The correlation between certain inflammatory biomarkers and hepatic steatosis has been explored and validated by multiple scholars. For instance, Zhao et al. (27) discovered a *U*-shaped association between the systemic immune-inflammation (SII) index and the risk of NAFLD, with an SII index of 422.40 serving as the inflection point. Zhou et al. (28) confirmed the non-linear association between the neutrophil-to-lymphocyte ratio (NLR) and the platelet-to-lymphocyte ratio (PLR) and NAFLD. Their investigation revealed PLR ≥ 42.29 potentially confer protective effects against NAFLD development, whereas NLR < 1.23 were identified as a risk factor of the disease. Overall, inflammation is a key factor in the onset of MAFLD. Immune mechanisms represent another key factor driving MAFLD progression. As Sutti et al. (29) reported in 2020, the recruitment of CD4+ Th1 and Th17 cells into the liver provides potent stimulation to hepatic macrophages, driving disease progression. Furthermore, CD8+ T cells and NKT cells accelerate hepatic inflammation and hepatocyte damage through interactions with liver cells (30). Albumin, synthesized in the liver, possesses anti-inflammatory and antioxidant properties; lower albumin levels signify a higher risk of hepatic metabolic dysregulation (31). Based on existing research, inflammation, nutritional status, and immune function significantly influence the development and progression of MAFLD (32). Consequently, detecting relevant indicators may be used to assess the odds of MAFLD. Clinically, early-stage MAFLD is often asymptomatic, and a single biomarker often falls short in clinical detection, making early screening for MAFLD somewhat challenging. Researchers have explored several combinations of biomarkers for MAFLD prediction. For instance, He et al. (32) investigated the association between the neutrophil-albumin ratio and MAFLD. Lu et al. (33) identified a non-linear relationship between PHR and NAFLD progression, including hepatic steatosis and fibrosis. Therefore, we chose to utilize composite markers in the hope of achieving superior efficacy in predicting MAFLD odds.

The CALLY index can reflect the overall inflammatory, immune, and nutritional status of the subjects. It was initially proposed by Iida et al. (34) in 2023 as a predictive biomarker for the post-operative prognosis of hepatocellular carcinoma. Now the CALLY index has gained recognition as a novel biomarker with prognostic utility for cardiovascular, cerebrovascular, and gastrointestinal conditions. We note that relevant indicators constituting of the CALLY index are readily obtainable in clinical work and are all closely associated to liver disease progression, but their potential association with MAFLD odds remains unexplored. To investigate this correlation and ensure the reliability of the results, we chose to conduct a joint analysis of public databases and clinical data. The analysis results of the three cohorts show consistency, revealing a significant inverse association between the ln-CALLY index and MAFLD prevalence. Comprehensive confounder adjustment strengthened these findings' validity. The inverse association persisted in models adjusting for covariates. Subgroup analyses and the RCS curve further validated the robustness of the results. Additionally, older age, men, specific race/ethnicity, obesity, and metabolic syndrome were associated with MAFLD, which is consistent with previous research (35). Notably, subgroup analyses revealed significant effect modification by sex in the UK Biobank cohort, whereas no such interaction was observed in the NHANES or Taizhou Central Hospital cohorts. Several factors may account for this discrepancy. First, the UK Biobank cohort is substantially larger than the NHANES and Taizhou Central Hospital cohorts, providing greater statistical power to detect modest interaction effects. Second, the UK Biobank population is pre-dominantly of European ancestry and comprises individuals aged 40 years and older, with a relatively balanced sex distribution, whereas the NHANES cohort includes a broader age range and more diverse racial/ethnic composition, and the Taizhou Central Hospital cohort consists of hospitalized patients with potentially different disease severity profiles.

Our study has several strengths. A primary strength of this research lies in its utilization of three independent, large-scale datasets from distinct populations across North America, Europe, and Asia (NHANES, UK Biobank, and a Chinese hospital cohort), thereby minimizing selection bias. The selected biomarkers for research have the advantage of low cost and are easily obtainable in clinical settings, making them a highly practical and potentially cost-effective tool for stratifying individuals more likely to have MAFLD in different clinical environments. Furthermore, the study employed a stringent analytical approach, including the use of successive multivariable logistic regression models to adjust for potential confounding variables, RCS regression to validate a non-linear dose-response relationship, and extensive subgroup analyses to examine the robustness of the association. These analytical strategies collectively enhanced the depth and credibility of the findings. However, there are still certain limitations in our study. Firstly, as a cross-sectional design, the causal relationship between the CALLY index and MAFLD remains unclear. Secondly, due to the high prevalence of MASLD in our study population, the odds ratios reported here may overestimate the true prevalence ratios. Readers should interpret the magnitude of associations with this in mind, and future studies may consider using log-binomial or modified Poisson regression to confirm these findings. Thirdly, MAFLD development is influenced by numerous factors, and unknown confounders may still impact the results. As a potential non-invasive biomarker, the predictive accuracy of the CALLY index requires further validation through large-scale prospective clinical studies. Additionally, the diagnostic criteria for hepatic steatosis differed between cohorts (CAP in NHANES and Taizhou Central Hospital, FLI in UK Biobank). Although both are validated non-invasive methods, they measure different aspects (direct physical attenuation vs. anthropometric and metabolic parameters) and may not be perfectly comparable, which may influence the final results. Finally, the ln-CALLY index achieved modest discriminative ability for identifying prevalent MAFLD (AUC = 0.650, 95% CI: 0.636–0.665), but its discriminative performance fell below the recommended threshold for clinical implementation (AUC > 0.800), indicating significant potential for further refinement, although the predictive performance of the ln-CALLY index was comparable to that of the aggregate index of systemic inflammation (AISI) and superior to other inflammatory indicators such as the NLR and PLR (36). Since the initiation of this study, the nomenclature for fatty liver disease has evolved, with metabolic dysfunction-associated steatitis liver disease (MASLD) now recommended as the preferred term. Although our study was designed using the MAFLD framework, the substantial overlap between MAFLD and MASLD populations supports the translatability of our findings. Given that the CALLY index components—CRP, albumin, and lymphocytes—reflect inflammation, nutrition, and immunity, all central to disease pathophysiology, we anticipate the observed inverse association would similarly apply to MASLD. Future studies adopting the MASLD framework are warranted to validate our findings within the updated nomenclature. Considering that MAFLD is a multifactorial disease, which encompasses key clinical parameters such as blood lipid profiles, glycemic status, and body mass index, the development of a composite predictive model integrating the CALLY index is anticipated to yield enhanced prognostic accuracy.

## 5 Conclusion

Our study demonstrates a robust inverse association between the CALLY index and the prevalence of MAFLD across three ethnically diverse cohorts. As relevant indicators constituting of the CALLY index are readily obtainable and inexpensive in clinical work, this biomarker shows promising potential as a predictive tool for stratifying individuals with elevated MAFLD susceptibility.

## Data Availability

The datasets presented in this study can be found in online repositories. The names of the repository/repositories and accession number(s) can be found below: the NHANES data was sourced from the NHANES public database, and the original data can be obtained at the website https://wwwn.cdc.gov/nchs/nhanes/default.aspx. The UK Biobank data can be obtained at the website https://www.ukbiobank.ac.uk/. Due to ethical limitations, data from Taizhou Central Hospital is not publicly available unless the researcher has legitimate reasons for requesting access and obtains the consent of the corresponding author.
