# Supplementary material for: The C-reactive protein-albumin-lymphocyte index: a novel biomarker for metabolic dysfunction-associated fatty liver disease across three ethnic cohorts
Source: Front Public Health. 2026 Apr 10;14:1737437. doi: 10.3389/fpubh.2026.1737437 (PMC13105867; doi:10.3389/fpubh.2026.1737437)
Supplement: Supplementary file 1 [file Table_1.docx]

**Supplementary table.** Association between ln-CALLY and the risk of MAFLD in UK Biobank.

|  | **Model 1** | | | **Model 2** | | | **Model 3** | | |
| --- | --- | --- | --- | --- | --- | --- | --- | --- | --- |
| **Characteristic** | **OR1** | **95% CI1** | **p-value** | **OR1** | **95% CI1** | **p-value** | **OR1** | **95% CI1** | **p-value** |
| **ln-CALLY** | 0.5498 | 0.5498, 0.5460 | **<0.001** | 0.6614 | 0.6556, 0.6671 | **<0.001** | 0.6731 | 0.6669, 0.6794 | **<0.001** |
| **ln-CALLY quartile** |  |  |  |  |  |  |  |  |  |
| Q1 | — | — |  | — | — |  | — | — |  |
| Q2 | 0.6172 | 0.6060, 0.6286 | **<0.001** | 0.7583 | 0.7399, 0.7771 | **<0.001** | 0.7426 | 0.7235, 0.7622 | **<0.001** |
| Q3 | 0.3671 | 0.3603, 0.3741 | **<0.001** | 0.5174 | 0.5046, 0.5305 | **<0.001** | 0.5173 | 0.5037, 0.5313 | **<0.001** |
| Q4 | 0.1628 | 0.1594, 0.1662 | **<0.001** | 0.2737 | 0.2663, 0.2812 | **<0.001** | 0.2925 | 0.2841, 0.3012 | **<0.001** |

Model 1: No covariates were adjusted. Model 2: Age, sex, race, education, TDI, BMI, smoking, and drinking were adjusted. Model 3: Age, sex, race, education, TDI, BMI, smoking, drinking, hypertension, diabetes, ALT, AST and serum creatinine were adjusted. ^1^OR = Odds Ratio, CI = Confidence Interval,TDI = Townsend deprivation index
